# β-Caryophyllene, a CB2-Receptor-Selective Phytocannabinoid, Suppresses Mechanical Allodynia in a Mouse Model of Antiretroviral-Induced Neuropathic Pain

**DOI:** 10.3390/molecules25010106

**Published:** 2019-12-27

**Authors:** Esraa Aly, Maitham A. Khajah, Willias Masocha

**Affiliations:** Department of Pharmacology and Therapeutics, Faculty of Pharmacy, Kuwait University, Safat 13110, Kuwait; esraa.m.aly89@gmail.com (E.A.); maitham@hsc.edu.kw (M.A.K.)

**Keywords:** neuropathic pain, nucleoside reverse transcriptase inhibitor, cytokines, ddC, phytocannabinoid, β-caryophyllene, antiretroviral, mechanical allodynia, CB2 receptor

## Abstract

Neuropathic pain associated with nucleoside reverse transcriptase inhibitors (NRTIs), therapeutic agents for human immunodeficiency virus (HIV), responds poorly to available drugs. Smoked cannabis was reported to relieve HIV-associated neuropathic pain in clinical trials. Some constituents of cannabis (*Cannabis sativa*) activate cannabinoid type 1 (CB1) and cannabinoid type 2 (CB2) receptors. However, activation of the CB1 receptor is associated with side effects such as psychosis and physical dependence. Therefore, we investigated the effect of β-caryophyllene (BCP), a CB2-selective phytocannabinoid, in a model of NRTI-induced neuropathic pain. Female BALB/c mice treated with 2′-3′-dideoxycytidine (ddC, zalcitabine), a NRTI, for 5 days developed mechanical allodynia, which was prevented by cotreatment with BCP, minocycline or pentoxifylline. A CB2 receptor antagonist (AM 630), but not a CB1 receptor antagonist (AM 251), antagonized BCP attenuation of established ddC-induced mechanical allodynia. β-Caryophyllene prevented the ddC-induced increase in cytokine (interleukin 1 beta, tumor necrosis factor alpha and interferon gamma) transcripts in the paw skin and brain, as well as the phosphorylation level of Erk1/2 in the brain. In conclusion, BCP prevents NRTI-induced mechanical allodynia, possibly via reducing the inflammatory response, and attenuates mechanical allodynia through CB2 receptor activation. Therefore, BCP could be useful for prevention and treatment of antiretroviral-induced neuropathic pain.

## 1. Introduction

Antiretroviral combination therapy is used to treat human immunodeficiency virus (HIV) infection and has resulted in the lowering of viral load, minimized viral transmission and made HIV a chronic disease rather than a fatal one [1,2]. The introduction of highly-active antiretroviral therapy (HAART) dramatically increased HIV patients’ life expectancies. Unfortunately, this increase in life expectancy came with a price. All antiretroviral therapies possess side effects and toxicities, which range from mild to life-threatening toxicities [3]. The initial antiretroviral therapy (ART) regimen for a treatment of naïve patient generally consists of two nucleoside reverse transcriptase inhibitors (NRTIs) plus a drug from one of three drug classes: an integrase strand transfer inhibitor (INSTI), a non-nucleoside reverse transcriptase inhibitor (NNRTI) or a boosted protease inhibitor (PI) [4].

Zidovudine, a NRTI, was the first drug approved to treat HIV [3,5]. Since then, NRTIs have been the cornerstone for ART. However, some of the NRTIs cause a dose-limiting neuropathy which has a huge impact on compliance, adherence and patient quality of life. These neuropathies were mainly associated with the dideoxynucleoside reverse transcriptase inhibitors (D drugs), which include zalcitabine (2′-3′-dideoxycytidine, ddC), didanosine (ddI) and stavudine (d4T) [6,7,8]. Moreover, the HIV virus produces distal symmetric polyneuropathy (HIV-DSP) which cannot be clinically distinguished from the antiretroviral toxic neuropathy (ATN) [7,9]. 

The clinical picture for both neuropathies involves a combination of negative (e.g., loss of sensation, hypoesthesia, hypoalgesia) and positive sensory symptoms (e.g., spontaneous pain, evoked pain, allodynia, hyperalgesia). Pain arises gradually, typically described as aching or numbness, and is characterized by a classical distal “glove and stocking” distribution as symptoms occur mainly in feet and lower extremities followed by hands [8,10]. The most prominent pathological features include Wallerian distal axonal degeneration, neuronal loss in dorsal root ganglia (DRG) of affected nerves, inflammatory cell infiltration (especially macrophages), reduced epidermal nerve fiber (ENF) density and a ‘dying back’ sensory neuropathy [9,10].

Over the years several animal models using ddC as a representative NRTI have been established in an attempt to understand the pathophysiology or to find treatment options for ATN. The ddC-induced neuropathy is accompanied by mechanical allodynia [11], hyperalgesia [12], decreased conduction velocity in C fiber afferents [13] and upregulation of inflammatory molecules such as tumor necrosis factor alpha (TNF-α), stromal cell-derived factor (SDF1-α) [14], and chemokine receptor type 4 (CXCR4) [15], caspases [16], interleukin 1 beta (IL-1β) and Wnt5a [17]; ddC-induced neuropathy also is accompanied by mitochondrial dysfunction [18,19,20].

Despite several clinical trials, there is no Food and Drug Administration (FDA) approved medication for either prevention or treatment of ATN [21]. The regular pharmacological treatments for other forms of neuropathic pain are widely used, although not proven effective in patients with HIV-associated neuropathic pain. These include antidepressants [22,23], anticonvulsants [24,25], topical agents, nonsteroidal anti-inflammatory drugs (NSAIDs) and opioids [6,26,27]. Smoked cannabis [28,29] and the capsaicin 8% transdermal patch [30] have proven to be effective against HIV-associated neuropathic pain in randomized clinical trials [27]. Despite these findings, it is well known that cannabis (*Cannabis sativa*) produces psychosis as a side effect; its use is prohibited in most countries and smoking carry significant health risks [31,32].

Recently, Munawar et al., reported the involvement of the endocannabinoid system in ddC-induced neuropathy and that the endocannabinoids anandamide (AEA) and 2-arachidonoylglycerol (2-AG) have anti-hyperalgesic activity via both cannabinoid type 1 (CB1) and cannabinoid type 2 (CB2) receptors [12]. The facts that cannabis reduced HIV sensory neuropathy (HIV-SN) and that endocannabinoids reduced ATN all lead to the supposition that a CB2 receptor agonist that does not produce psychosis as a side effect would be a good candidate to prevent or treat ATN.

β-Caryophyllene (BCP) is a phytocannabinoid found in essential oils of various plants including black pepper (*Piper nigrum*), cinnamon (*Cinnamomum* spp.), lemon balm (*Melissa officinalis*), cloves (*Syzygium aromaticum*), oregano (*Origanum vulgare*), hops (*Humulus lupulus*) and cannabis (*Cannabis sativa*) [33,34]. It is approved to be used as a natural flavoring agent by the FDA [35]. β-Caryophyllene is a CB2-receptor-selective agonist [33] that produces antinociception but lack the psychotic side effects produced through CB1 receptors [36]. It has pleiotropic activities including antioxidant [37], anti-inflammatory [38], neuroprotective [39], anxiolytic [40], anticancer and analgesic effects [41]. In addition, it attenuated neuropathic pain in several models including models of chemotherapy-induced neuropathy [42], diabetic neuropathy [43], sciatic nerve partial ligation [36] and chronic constriction injury of the sciatic nerve [44,45].

In this study, we investigated whether concomitant treatment with BCP and ddC can prevent the development of ddC-induced neuropathic pain and inflammation. In addition, we compared the antiallodynic effects of BCP to minocycline and pentoxifylline, which have been shown to have anti-inflammatory and antiallodynic effects against various types of neuropathic pain [46,47,48,49].

## 2. Results

### 2.1. Effect of ddC on Withdrawal Threshold to Mechanical and Thermal Stimulation

**Figure 1:**
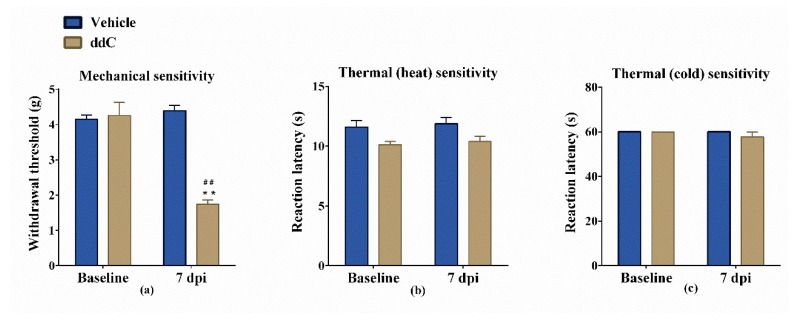
Effects of 2′-3′-dideoxycytidine (ddC) on sensitivity of female BALB/c mice to mechanical and thermal (hot and cold) stimuli. (**a**) Withdrawal threshold to the dynamic plantar aesthesiometer before (baseline) and at day 7 post-first-injection (7 dpi) of ddC. (**b**) Reaction latency to the hot plate (55 °C) before and at 7 dpi of ddC. (**c**) Reaction latency to the cold plate (4 °C) before and at 7 dpi of ddC. Each bar represents the mean ± SEM of values obtained from eight animals. ** *p* < 0.01 compared to vehicle-treated control mice at the same day after treatment (two-way repeated measures ANOVA followed by Sidak’s multiple comparisons test); ## *p* < 0.01 compared to pretreatment baseline values (Mann–Whitney test).

Administration of ddC 25 mg/kg i.p. for 5 days produced a significant reduction in withdrawal threshold to mechanical stimuli (mechanical allodynia) at day 7 post first ddC injection compared to baseline values (1.742 ± 0.122 g versus 4.258 ± 0.3715 g, *p* < 0.01) and to vehicle-treated control mice at day 7 (mean 1.742 ± 0.122 g compared to 4.392 ± 0.1580 g, *p* < 0.01) (Figure 1a). On the other hand, administration of ddC did not modulate the sensitivity to either hot or cold stimuli (Figure 1b,c).

### 2.2. β-Caryophyllene, Minocycline and Pentoxifylline Prevent the Development of ddC-Induced Mechanical Allodynia

The administration of BCP, minocycline or pentoxifylline 16 h before first administration of ddC and concomitantly with ddC for 5 days, significantly prevented the development of ddC-induced mechanical allodynia at day 7 post-first-administration of ddC.

**Figure 2:**
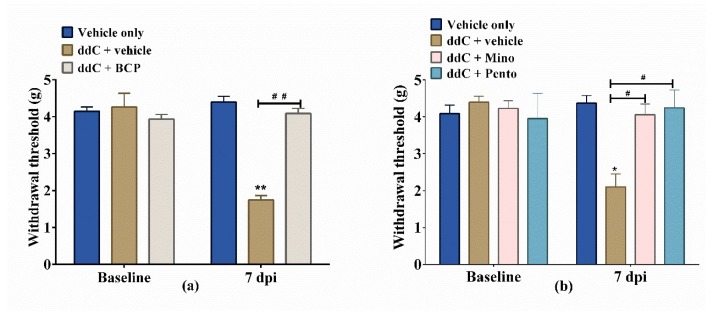
β-Caryophyllene (BCP), minocycline (Mino) and pentoxifylline (Pento) prevent the development of 2′-3′-dideoxycytidine (ddC)-induced mechanical allodynia in female BALB/c mice. The effect of treatment with (**a**) BCP, (**b**) minocycline and pentoxifylline on the development of ddC-induced mechanical allodynia at day 7 post-first-injection (dpi) of ddC. Each bar represents the mean ± SEM of values obtained from seven to eight animals. * *p* < 0.05, ** *p* < 0.01 compared to vehicle-only-treated control mice at day 7, and # *p* < 0.05, ## *p* < 0.01 compared to mice treated with ddC + vehicle (two-way repeated measures ANOVA followed by Sidak’s multiple comparisons test).

Treatment with ddC significantly reduced the withdrawal threshold of mice to the dynamic plantar aesthesiometer on day 7 compared to vehicle-only-treated control mice, with values of 1.74 ± 0.12 g versus 4.39 ± 0.16 g, respectively (*p* < 0.01; Figure 2a). On the other hand, mice treated with ddC plus BCP had withdrawal threshold similar to the vehicle-only-treated control mice, with values of 4.09 ± 0.14 g versus 4.39 ± 0.16 g, respectively (*p* > 0.05), which were significantly higher than those of the mice treated with ddC plus vehicle (*p* < 0.01; Figure 2a). Mice treated with ddC plus minocycline (4.06 ± 0.30 g) or pentoxifylline (4.24 ± 0.18 g) had withdrawal thresholds similar (*p* > 0.05) to the vehicle-only-treated control mice, (4.37 ± 0.21 g) which were significantly higher than those of the mice treated with ddC plus vehicle (2.10 ± 0.21 g; *p* < 0.01; Figure 2b).

### 2.3. β-Caryophyllene Attenuates Established ddC-Induced Mechanical Allodynia in a CB2-Receptor-Dependent Manner

**Figure 3:**
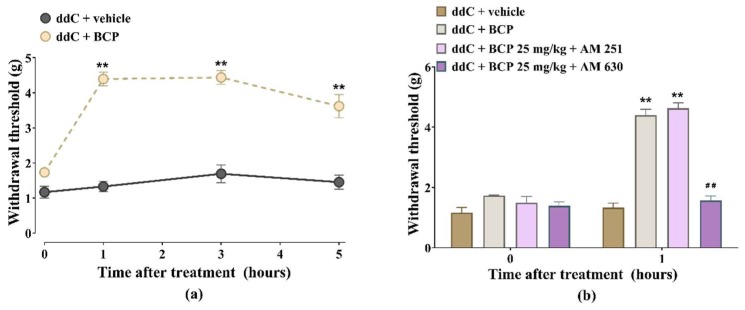
Antiallodynic effects of β-caryophyllene (BCP) against established 2′-3′-dideoxycytidine (ddC)-induced mechanical allodynia in female BALB/c mice is antagonized by CB2, but not CB1, receptor antagonist. (**a**) Acute antiallodynic effects of BCP 25 mg/kg on mice with established ddC-induced mechanical allodynia. β-Caryophyllene was administered at day 7 post-first-injection of ddC. Mechanical sensitivity was measured by dynamic plantar aesthesiometer. Each point represents the mean ± SEM of values obtained from four animals. ** *p* < 0.01 compared to mice treated with ddC + vehicle. (**b**) Effects of AM 251, a CB1 receptor antagonist, and AM 630, a CB2 receptor antagonist, on the antiallodynic effects of BCP on mice with ddC-induced mechanical allodynia 1 h after administration. Each bar represents the mean ± SEM of values obtained from four animals. ** *p* < 0.01 compared to mice treated with ddC + vehicle and ## *p* < 0.01 compared to mice treated with ddC + BCP (two-way repeated measures ANOVA followed by Sidak’s multiple comparisons test).

Treatment of mice with ddC-induced mechanical allodynia with BCP 25 mg/kg resulted in an increase in withdrawal threshold to mechanical stimuli (antiallodynic effects) at all-time points, from 1 to 5 h (*p* < 0.01; Figure 3a).

The CB1 receptor antagonist AM 251 did not significantly affect the antiallodynic effect of BCP, i.e., there was no difference in withdrawal threshold between mice treated with BCP alone (4.4 ± 0.2 g) and those treated with BCP + AM 251 4.6 ± 0.2 g (*p* > 0.05; Figure 3b), whereas the CB2 receptor antagonist AM 630 significantly prevented the antiallodynic effect of BCP, i.e., reduction in withdrawal threshold from 4.4 ± 0.2 g for BCP alone to 1.6 ± 0.1 g for BCP + AM 630 (*p* < 0.05; Figure 3b).

### 2.4. β-Caryophyllene Prevents the ddC-Induced Upregulation of Proinflammatory Cytokine Transcripts in the Paw Skin and Brain

**Figure 4:**
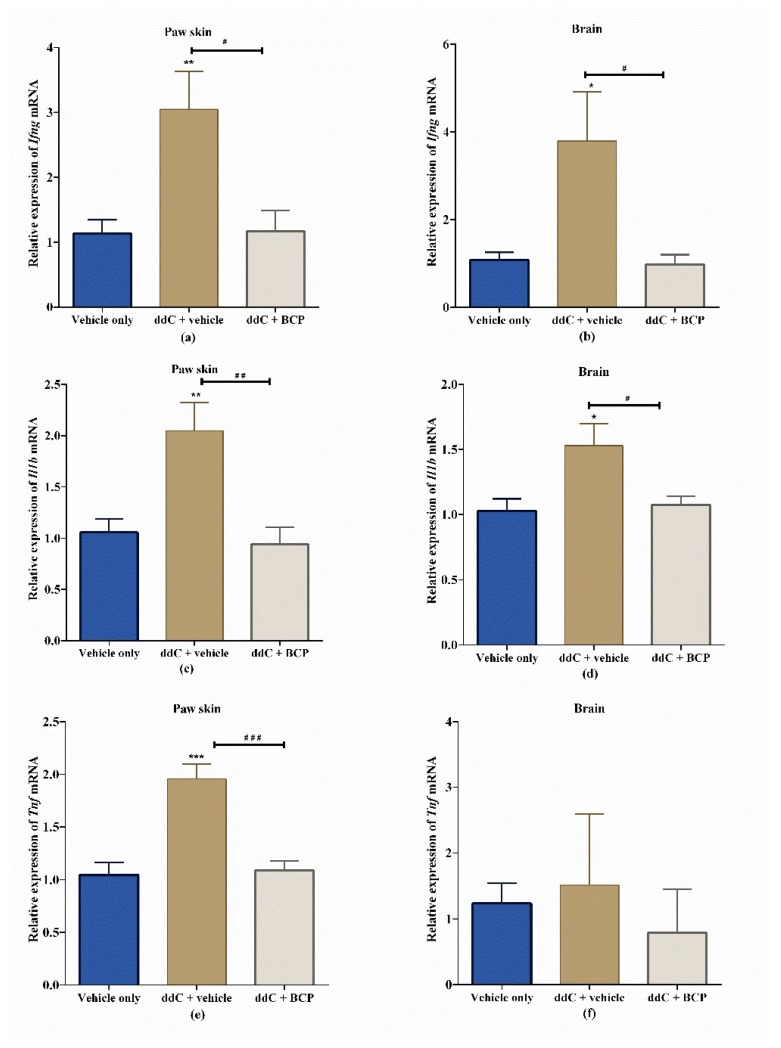
Effect of treatment with 2′-3′-dideoxycytidine (ddC) or ddC plus β-caryophyllene (BCP) on the relative expression of proinflammatory cytokine mRNA in paw skins and brains of female BALB/c mice at day 7 post first ddC dose. Expression of mRNA of (**a**) interferon gamma (*Ifng*) in the paw skin, (**b**) *Ifng* in the brain, (**c**) interleukin 1 beta (*Il1b*) in the paw skin, (**d**) *Il1b* in the brain, (**e**) tumor necrosis factor alpha (*Tnf*) in the paw skin and (**f**) *Tnf* in the brain, on day 7 post-first-injection of ddC. Each bar represents the mean ± SEM of values, except for *Tnf* in the brain (median and interquartile range), obtained from seven to eight animals. * *p* < 0.05, ** *p* < 0.01, *** *p* < 0.001 compared to control mice (treated with vehicle only) at day 7; # *p* < 0.05, ## *p* < 0.01, ### *p* < 0.001 compared to mice treated with ddC plus vehicle. For (**a**–**e**) one-way ANOVA was followed by Tukey’s multiple comparisons test and for (**f**) Kruskal–Wallis was followed by Dunn’s multiple comparisons test.

Treatment with ddC significantly increased the expression of interferon gamma (*Ifng*) (Figure 4a,b) and interleukin-1 beta (*Il1b*) (Figure 4c,d) mRNA in both the paw skin and the brain compared to vehicle-only-treated control mice (*p* < 0.05). The levels of tumor necrosis factor (*Tnf*) transcripts were significantly upregulated in the paw skin (*p* < 0.001; Figure 4e) but not in the brain (*p* > 0.05; Figure 4f) of ddC-treated mice compared to vehicle-only-treated control mice. Interestingly, coadministration of ddC with BCP significantly prevented the ddC-induced upregulation of inflammatory cytokines’ mRNA transcripts, i.e., the transcript levels of cytokines in the paw skins and brains of mice treated with ddC plus BCP were significantly lower than those of mice treated with ddC plus vehicle (*p* < 0.05). The relative expression of inflammatory cytokine mRNA for mice cotreated with BCP and ddC were similar to vehicle-only-treated control mice (*p* > 0.05; Figure 4).

### 2.5. ddC Does Not Alter Glial Cell Markers in the Brain

**Figure 5:**
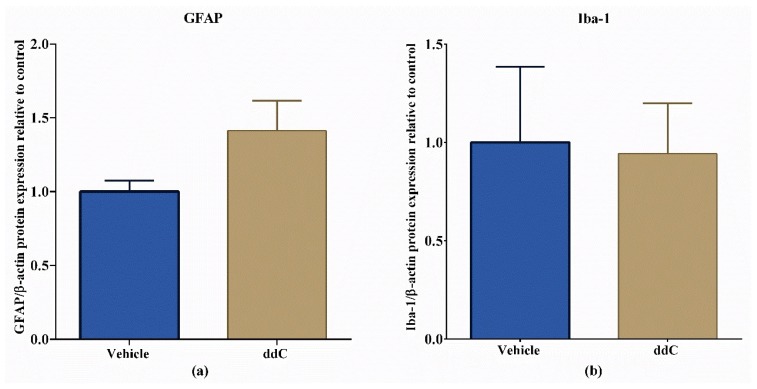
Effect of treatment with 2′-3′-dideoxycytidine (ddC) on protein expression of ionized calcium binding adaptor molecule 1 (Iba-1) and glial fibrillary acidic protein (GFAP) in the brains of female BALB/c mice at day 7 post-first-injection (dpi) of ddC. Relative expression of (**a**) GFAP and (**b**) Iba-1 in the brain. The protein expression was measured using the Wes^TM^ capillary-based protein electrophoresis. The ratio between the area of the electropherogram of the protein of interest and the area of β-actin were calculated and normalized to the control group. The bars represent the mean ± SEM of values obtained from eight animals for GFAP and median and interquartile range of values obtained from four animals for Iba-1.

Treatment with ddC had no significant effect on the protein levels of GFAP, an astrocyte marker, and Iba-1, a microglia marker, in the brain compared to vehicle-only-treated control mice (*p* > 0.05; Figure 5).

### 2.6. β-Caryophyllene Prevents the ddC-Induced Phospho-Erk1/2 Levels in the Brain

**Figure 6:**
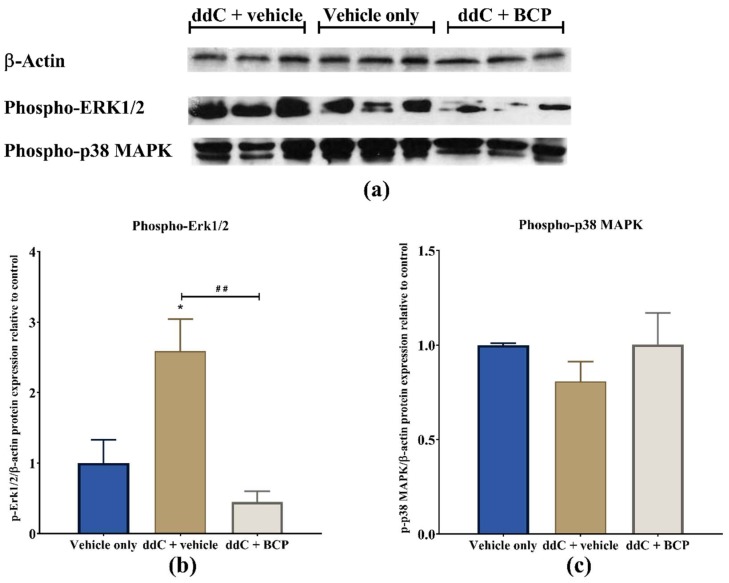
Effect of treatment with 2′-3′-dideoxycytidine (ddC) or coadministration of ddC with β-caryophyllene (BCP) on the levels of phosphorylated p44/42 MAPK (phospho-extracellular-signal-regulated kinases 1 and 2, p-Erk1/2), and phosphorylated p38 mitogen-activated protein kinases (p-p38 MAPK) in the brains of female BALB/c mice at day 7 post-first-injection (dpi) of ddC. (**a**) X-ray images of the blots; (**b**) relative expression of p-Erk1/2; (**c**) relative expression of p-p38 MAPK. Their expression profile was normalized to β-actin and the control group. The bars represent the mean ± SEM of values obtained from three animals. * *p* < 0.05 compared to vehicle-only-treated control mice, and ## *p* < 0.01 compared to mice treated with ddC + vehicle (one-way ANOVA was followed by Tukey’s multiple comparisons test).

Treatment with ddC significantly increased the amount of phosphorylated extracellular-signal-regulated kinases 1 and 2 (p-Erk1/2) (*p* < 0.05; Figure 6a,b), but not phosphorylated p38 mitogen-activated protein kinases (p-p38 MAPK; *p* > 0.05; Figure 6a,c), in the brain compared to vehicle-only-treated control mice. Coadministration of ddC with BCP significantly prevented the ddC-induced increase in the levels of phosphorylated Erk1/2 (*p* < 0.01; Figure 6a,b).

## 3. Discussion

The results of the current study show that treatment of mice with ddC induced mechanical allodynia, did not change thermal sensitivity and elevated the levels of proinflammatory cytokine (*Ifng*, *Il1b* and *Tnf*) mRNA in both their brains and paw skins. Treatment with ddC increased the level of phospho-Erk1/2 but did not alter the levels of phospho-p38 MAPK or glial cell markers GFAP and Iba-1. Cotreatment with BCP prevented the development of ddC-induced mechanical allodynia and ddC-induced upregulation of proinflammatory cytokine transcripts and phospho-Erk1/2. Cotreatment with minocycline or pentoxifylline also prevented the development of ddC-induced mechanical allodynia. Acute treatment with BCP attenuated already-established mechanical allodynia. The antiallodynic effect of BCP was prevented by a CB2, but not a CB1, receptor antagonist.

Treatment with ddC induced mechanical allodynia in mice similar to what has been reported previously in mice treated with different regimens of ddC [17,50,51]. These findings concur with what was observed in studies on patients with HIV-associated neuropathic pain where some patients had mechanical allodynia [52,53]. The mice did not have changes in thermal hypersensitivity, similar to what was described by Wallace et al. where ddC induced mechanical allodynia but did not alter the response to thermal stimuli in rats [54]. However, this is in contrast to our previous findings where a single dose of ddC induced mechanical allodynia and thermal hyperalgesia [12,50], possibly because of the difference in number of administrations and cumulative dose, i.e., five administrations with a cumulative dose of ddC of 125 mg/kg compared to a single dose of 25 mg/kg in the previous studies.

The increase in the levels of cytokine transcripts in the paw skin and brains of mice occurred at the time point when they had developed ddC-induced mechanical allodynia. However, ddC did not alter the levels of the markers for astrocytes and microglia, GFAP and Iba-1, respectively, in the brain. This suggests that astrocytes and microglia are not the source of cytokines in the brains of female mice with ddC-induced allodynia. However, in male rodents, ddC was found to induce astrocyte and microglia activation in the spinal cord [17,54,55]. The differences in our findings and these previous studies are possibly due to that we used female rodents, while they used male rodents, and we studied the brain, while they studied the spinal cord. Sex/gender differences in the manifestation and mechanisms involved in pain have been described and microglia have been suggested to be important for neuropathic pain in male rodents, but not female rodents, while T cells could be important for neuropathic pain in female rodents [56,57,58]. Further research is needed to find the source of the cytokines both in the periphery and the central nervous system (CNS) of female mice. Elevated levels of proinflammatory cytokines such as TNF-α, IFN-γ and IL-1β have been associated with various types of neuropathic pain in both humans and animal models, and inhibitors or antagonists of some these cytokines have been reported to alleviate allodynia and pains scores [59,60,61,62,63,64,65]. Treatment-naïve HIV infected patients have been reported to have increased cytokine levels in the plasma soon after initiation of ART [66]. The HIV patients who developed neuropathic pain after receiving ART were found to have altered cytokine levels even before ART treatment [66]. Treatment of rats with ddC resulted in increased levels of both transcripts and proteins of TNF-α in the spinal cord and dorsal root ganglion (DRG) neurons at a time point when the rats had developed mechanical allodynia [55]. Aging mice treated with ddC had increased TNF-α and IL-1β proteins in the spinal cord [17]. These studies and our current findings suggest that proinflammatory cytokines both in the periphery and the CNS play a role in the pathophysiology of antiretroviral drug-induced neuropathic pain. The active role of the proinflammatory cytokine in ddC-induced mechanical allodynia was validated by the fact that both knockdown of TNF-α with intrathecal siRNA and administration of recombinant TNF soluble receptor, which neutralizes the biological effect of TNF-α, reversed the ddC-induced mechanical allodynia [55].

The family of MAPKs, including p38 MAPK, Erk1/2 and c-Jun N-terminal kinase (c-JNK), has been implicated in excessive sensitization of sensory neurons and neuropathic pain [67,68]. The activation of p38 MAPK has a prominent role in inflammatory responses and the activation of microglia is accompanied by activation (phosphorylation) of p38 MAPK [67]. In the current study, the fact the ddC did not alter the levels of phosphorylated p38 MAPK correlates with the lack of microglia activation in the brain after ddC treatment. Our results are in agreement with those of Huang et al., who reported no significant difference in p-p38 MAPK activation in microglia in the dorsal horn in response to stavudine (NRTI) [69]. However, they differ with those of Zheng et al., who observed that HIV gp120 combined with ddC induced the upregulation of phospho-p38 MAPK in the spinal cord dorsal horn [70]. The difference between our findings and those of Zheng et al. could be due to various reasons including that they used HIV gp120 in combination with ddC in male rats and evaluated phospho-p38 MAPK in the spinal cord, while we used ddC alone in female mice and evaluated phospho-p38 MAPK in the brain. In the current study, mice with ddC-induced mechanical allodynia had elevated levels of phorpho-Erk1/2. The upregulation of phospho-Erk1/2 has been linked to the pathogenesis of pain both in the peripheral nervous system and CNS [71]. However, to our knowledge this is the first study to report the expression of Erk1/2 in the CNS after antiretroviral drug treatment. In the periphery, the ratio of p-ERK1/2 to ERK1/2 was increased in the DRG of rats treated with HIV gp120 plus ddC [72]. However, peripheral (intradermal) administration of an Erk1/2 inhibitor was reported to have no effect on ddC-induced mechanical hypersensitivity [11]. Further studies on the role of Erk1/2 in antiretroviral drug-induced allodynia are warranted.

Drugs that have immunomodulatory effects and suppress the expression of cytokines and signaling molecules such as phospho-Erk1/2 could be useful in alleviating antiretroviral drug-induced mechanical allodynia.

β-Caryophyllene is a phytocannabinoid, which is a CB2-receptor-selective agonist [33]. It has been shown to reduce the expression of cytokines and attenuate mechanical allodynia in animal models of paclitaxel-induced neuropathic pain and diabetic neuropathic pain [42,43]. It has also been shown to decrease elevated levels of phospho-Erk1/2 in disease models of inflammation [33,73]. In the current study, BCP suppressed the expression of the transcripts of cytokines, the levels of phospho-Erk1/2 and mechanical allodynia in a mouse model of antiretroviral drug-induced neuropathic pain. The findings from these studies and the current study suggest that BCP inhibition of neuropathic-pain-induced elevation of cytokine expression and phospho-Erk1/2 could be one of its mechanisms in preventing the development of mechanical allodynia. These studies and the findings of the current study suggest that one of the mechanisms BCP utilizes to prevent the development of mechanical allodynia is possibly through suppression of diabetes, paclitaxel or NRTI-induced elevation of cytokine expression and phospho-Erk1/2. The antiallodynic effects of BCP have been reported to be via activation of CB2 receptors [36,42]. In the current study, BCP attenuated the ddC-established mechanical allodynia. The antiallodynic effects of BCP were antagonized by a CB2, but not a CB1, receptor antagonist, similar to what has been described previously against paclitaxel-induced allodynia [42]. The CB2 receptor was isolated from human myeloid cell line HL-60 [74], is expressed abundantly in immune and neuroimmune cells and its activation has been shown to result mainly in anti-inflammatory activities including the reduction in expression of proinflammatory cytokines in astrocytes, microglia, macrophages, dendritic cells, T cells and other immune cells [75,76,77]. Thus, BCP suppressed the ddC-induced upregulation in proinflammatory cytokine expression and mechanical allodynia possibly through activation of CB2 receptors and downregulation of the levels of phosphorylated ERK1/2.

The antiallodynic activities of BCP against ddC-induced mechanical allodynia were found to be similar to those of other immunomodulators, pentoxifylline and minocycline. However, BCP has the advantage of also directly attenuating established mechanical allodynia via CB2 receptors, whereas previous preclinical studies show that minocycline cannot attenuate established hyperalgesia and allodynia [78,79,80], which is possibly one of the reasons we postulated [50] for its failure in clinical trials for neuropathic pain [81]. Moreover, BCP alleviates allodynia in both female (current study and in another study [43]) and male mice [42], whereas a recent study showed that minocycline and pentoxifylline could reverse mechanical thresholds in males, but not in female mice [82]. Treatment with drugs that prevent astrocyte and microglia activation has been found to prevent or reduce neuropathic pain in various models [56,83,84,85]. Pentoxifylline, a glial inhibitor, has been shown to inhibit inflammatory cytokine production in various models of neuropathic pain [86,87] and to reduce ddC-induced mechanical allodynia in rats [55]. Similarly, in the current study pentoxifylline prevented the development of ddC-induced mechanical allodynia in mice. Treatment with a microglia inhibitor, minocycline, which inhibits inflammatory cytokine production during neuropathic pain [49,88,89,90], also prevented the development of ddC-induced mechanical allodynia in mice. However, in a previous study minocycline did not prevent the development of ddC-induced mechanical allodynia in rats [54]. The discrepancy between the current study and that of Wallace et al. [54] could be due to differences in the gender and species of the experimental animals (female BALB/c mice versus male Wistar rats) as well as the doses of minocycline used (50 mg/kg versus 40 mg/kg).

In summary, ddC induced mechanical allodynia and upregulated proinflammatory cytokines both in the periphery and CNS of BALB/c mice. ddC also increased the levels of the signaling molecule phospho-Erk1/2, but not phospho-p38 MAPK. Treatment with BCP and other immunomodulatory drugs minocycline and pentoxifylline protected against the development of ddC-induced mechanical allodynia. The antiallodynic effects of BCP are dependent on CB2, but not CB1, receptors. β-Caryophyllene also suppressed the ddC-induced upregulation of proinflammatory cytokine transcripts both in the periphery and the CNS, as well as the ddC-induced increase in levels of phospho-Erk1/2. Thus, BCP prevented the development of ddC-induced mechanical allodynia possibly via its anti-inflammatory activities both in the periphery and CNS. Oral administration of BCP concomitantly with antiretroviral drugs could be of clinical value in preventing the development of antiretroviral drug-induced inflammation and neuropathic pain. β-Caryophyllene has the advantage of lacking the psychoactive effects of cannabis and being available as a natural substance already approved by the FDA as a flavoring agent.

## 4. Materials and Methods

### 4.1. Animals

Female BALB/c mice (8–12 weeks old) were provided by the Animal Resources Centre, Kuwait University, Kuwait. Animals were kept into controlled temperature of 24 ± 1 °C and had access to food and water ad libitum. All behavioral studies were conducted between 08:00 and 16:00 h to exclude diurnal variations. All experiments were approved by the Ethical Committee for the use of Laboratory Animals in Teaching and in Research, Health Sciences Centre (HSC) and were preformed according to Directive 2010/63/EU of the European Parliament and of the council on the protection of animals used for scientific purposes.

### 4.2. Drugs

After being dissolved in normal saline, 2′-3′-dideoxycytidine (ddC, zalcitabine) (Sigma Aldrich, St Louis, MO, USA) was injected intraperitoneally (i.p.) at a dose of 25 mg/kg in a volume of 10 mL/kg. β-Caryophyllene (BCP) (Sigma Aldrich) was mixed with normal saline and put in the sonicator until emulsion formation and administered by oral gavage at a loading dose of 50 mg/kg and a maintenance dose of 25 mg/kg twice daily for 5 days, or at a dose of 25 mg/kg once after neuropathic pain had been established, similar to the doses used previously [42]. Minocycline (Sigma Aldrich) was dissolved by adding normal saline followed by ultra-sonication until completely dissolved and was administered i.p. at a dose of 50 mg/kg, similar to the doses used previously [50]. Pentoxifylline (Sigma Aldrich) was dissolved in normal saline and was administered i.p. at a dose of 100 mg/kg, similar to the doses used previously [48]. The CB1 receptor antagonist AM 251 (Tocris, Bristol, UK) and the CB2 receptor antagonist AM 630 (Tocris) were dissolved (ultrasonicated to prevent foam formation) in normal saline containing 5% Tween 80 and 5% propylene glycol and both drugs were administered i.p. at a dose of 3 mg/kg, similar to the doses used previously [12,50,78]. All the drugs were freshly prepared on the day of administration.

### 4.3. Model of ddC-Induced Neuropathic Pain and Drug Treatment

For 5 days, 2′-3′-dideoxycytidine was injected intraperitoneally. Baseline withdrawal threshold to mechanical stimuli was assessed one day before the first ddC injection and the changes in mechanical sensitivity were assessed on day 7 post first ddC injection.

**Figure 7:**
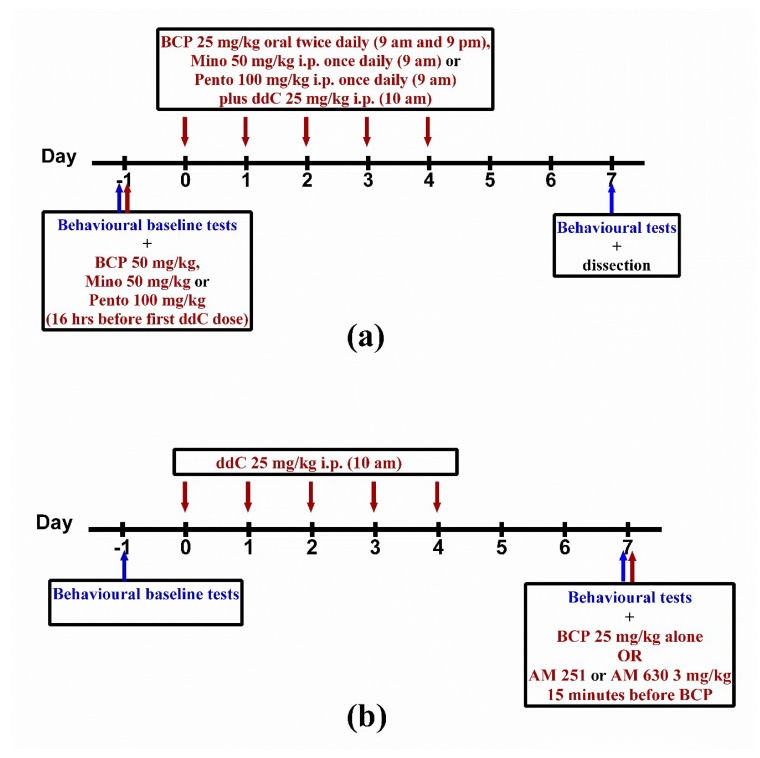
(**a**) Drug administration schedule for preventative treatment with β-caryophyllene (BCP), minocycline (Mino) or pentoxifylline (Pento) against 2′-3′-dideoxycytidine (ddC)-induced neuropathy. (**b**) Drug administration schedule for treatment with BCP against established ddC-induced neuropathy and for the CB receptor antagonists AM 251 and AM 630 before BCP. The maroon arrows indicate the days when the drugs were administered, while the blue arrows indicate when the behavioral tests were performed.

For preventative/prophylactic treatment, the loading dose of BCP (50 mg/kg) was administered by oral gavage 16 h before the first dose of ddC [91]. This was followed by a maintenance dose of 25 mg/kg twice daily at a 12-h interval for 5 days. In the morning, BCP was administered by oral gavage one hour before ddC injection (Figure 7a). Minocycline 50 mg/kg and pentoxifylline 100 mg/kg were injected i.p. 16 h before first ddC dose followed by once daily injection one hour before ddC for 5 days (Figure 7a).

In order to evaluate the effects of BCP on established mechanical allodynia, BCP (25 mg/kg) was administered once on day 7 after first dose of ddC (Figure 7b). AM 251 (3 mg/kg) and AM 630 (3 mg/kg) were administered 15 min before the administration of BCP in order to evaluate whether CB1 and/or CB2 receptors were involved in the antiallodynic effects of BCP.

### 4.4. Assessment of Mechanical Allodynia

Mechanical sensitivity was assessed using the dynamic plantar aesthesiometer (Ugo Basile, Gemonio, Italy), as described previously [50]. Briefly, each mouse was habituated in a plastic chamber on top of a mesh platform for one hour before assessment of response to mechanical stimuli. Mechanical stimuli were applied using a metal filament (0.5 mm diameter) with an increasing force (0.25 g/s) and the force at which the mice withdrew the hind paw was recorded. The cut-off force was 5 g. At least three different readings were recorded for each mouse.

### 4.5. Assessment of Response to Thermal Stimuli

Thermal sensitivity was assessed using the hot or cold plate (Panlab SL, Barcelona, Spain) at 55 ± 1 °C or 4 ± 1 °C, as described before [12,92,93]. Briefly, each mouse was placed individually in a hot or cold plate, and the first sign of nociception, paw licking, flinching or jump response to avoid the heat or cold was recorded and the animal immediately removed from the plate. Cut-off periods of 20 s for the hot plate and 60 s for the cold plate were maintained to avoid damage to the paws.

### 4.6. Disecction and Tissue Storage

Mice were anesthetized with halothane, sacrificed by decapitation on day 7 post-first-administration of ddC. Brains and paw skins were dissected; brains were separated by razor blade into two halves, immersed directly in liquid nitrogen and stored in −80 °C for RNA extraction and protein analysis.

### 4.7. Real Time RT-PCR

Total RNA from half brains and paw skins was extracted using the RNeasy Kit (Qiagen GmbH, Hilden, Germany), quantified using the NanoDrop 2000 spectrophotometer (Thermo Scientific, Wilminton, DE, USA) and reverse transcribed into cDNA, followed by quantification of mRNA levels on an ABI Prism^®^ 7500 sequence detection system (Applied Biosystems, Carlsbad, CA, USA) as described previously [94]. The primers for *Il1b*, *Tnf* and *Ifng* (primer sequence in Table 1) were purchased from Invitrogen Life Technologies. After obtaining the threshold cycle (Ct) values, the levels of mRNA for each sample were normalized to *Ppia* (cyclophilin A, housekeeping gene) ΔCt. Calculations of the relative amount of target gene transcripts were done using the 2^−ΔΔCt^ method by Livak and Schmittgen [95].

### 4.8. Western Blot

Half brains were added to a homogenization buffer (four times the weight of the brain) containing 50 mM HEPES, 50 mM NaCl, 5 mM EDTA, 1% Triton X, 10 µg/mL leupeptin, 10 µg/mL aprotinin, 100 µg/mL PMSF and deionized water, homogenized by sonication and centrifuged at 10,000 rpm at 4 °C for 15 min. The supernatant was transferred into a new Eppendorf tube and the protein concentrations were determined by the Bradford assay using bovine serum albumin (BSA) as standard. The brain homogenates were aliquoted and stored at −80 °C until use.

#### 4.8.1. Wes^TM^ Capillary-Based Protein Electrophoresis

**Figure 8:**
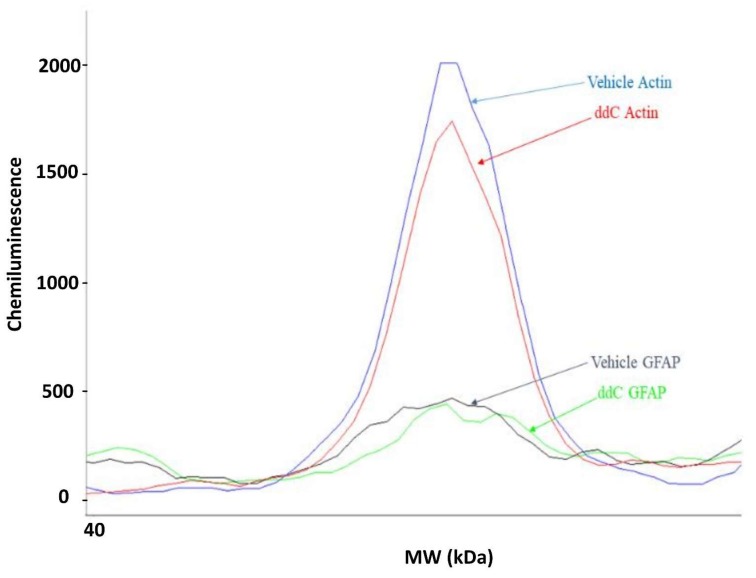
Electropherogram of actin and GFAP expression in brain samples from a control (vehicle-treated) and a ddC-treated mouse.

The GFAP and Iba-1, as well as the house keeping protein β-actin, protein levels in brain tissues were measured using the ProteinSimple Wes with the 12–230 kDa separation module (ProteinSimple, San Jose, CA, USA) following the manufacturer’s protocol. Briefly, brain homogenates were diluted to 1 mg/mL using 0.1× buffer. One part of 5× fluorescent mix was combined with four parts of the sample followed by denaturation at 95 °C for 5 min. The primary antibodies for rabbit anti-GFAP (Boster Bio, Pleasanton, CA, USA), rabbit anti-Iba-1 (Boster Bio) and rabbit anti-β-actin (Cell Signaling, Danvers, MA, USA) were diluted in a ratio of 1:50 using antibody diluent 2. The secondary anti-rabbit antibody was supplied with the kit (ProteinSimple). The 384-well assay plate was loaded according to the manufacturer’s instructions followed by centrifugation at 2500 rpm for 5 min at room temperature. The plate and the capillaries were loaded onto the Wes instrument, in which the separation electrophoresis and immunodetection steps take place in a fully automated manner. At the end of run, data analysis was done using the Compass Software (ProteinSimple). The resulting electropherograms (an example is shown in Figure 8) provided molecular weight, peak area, peak height and signal-to-noise ratio for each sample. The peak area represents the signal intensity of the immuno-detected protein. In this study, the ratios between the area of protein of interest and the area of β-actin were calculated and normalized to the control group. Data analysis was performed using a Microsoft Excel spreadsheet.

#### 4.8.2. Gel-Based Western Blot

The phospho-p38 MAPK and phospho-Erk1/2, as well as the house keeping protein β-actin, protein levels in brain tissues were measured using the traditional gel-based Western blot as previously described [96]. In order to denature proteins, the lysate (70 μL) was mixed with 28 μL of 5× Laemmli sample loading buffer (Tris base stock (0.5 M), glycerol, sodium dodecyl sulfate (SDS), 2-mercaptoethanol, bromophenol blue) followed by incubation in boiling water for 10 min at 90 °C. Samples were then centrifuged and stored at −20 °C for later usage. Samples (40 μg of protein) and ladder (Bio-Rad protein; Precision Plus Protein™ Dual Color Standards) were loaded onto 10% SDS-PAGE composed of a stacking gel to concentrate samples before they migrate into the separating gel. Samples were then electrophoresed at 90 V for 10 min followed by running at 120 V for 60 min. Proteins were transferred to a methanol-activated polyvinylidene difluoride (PVDF) membrane through electrophoresing at 95 V for 45 min. This was followed by a blocking step with 5% BSA for 1 h at room temperature. Subsequently, the membranes were incubated overnight at 4 °C with primary antibodies (all from Cell Signaling, Danvers, MA, USA) diluted according to manufacturer’s instructions, prepared in 5% BSA (β-actin, 1:1000; phospho-p38 MAPK (Thr180/Tyr182), 1:1000; and phospho-p44/42 MAPK (p-Erk1/2) (Thr202/Tyr204), 1:1000). The membranes were washed three times for 5 min with Tris base buffer saline—Tween (TBST)—and incubated with the secondary antibody (mouse anti-rabbit IgG-horseradish peroxidase (HRP), Santa Cruz, CA, USA) (1:1000 dilution, prepared in 5% BSA) for 45 min at room temperature. The membranes were washed three times for 5 min with TBST followed by a final wash of TBS for 5 min. Bands were developed with prime ECL (Amersham ECL Prime Western Blotting Detection Reagent, GE Life Sciences, Chicago, IL, USA) in the dark room and visualized with Santa Cruz X-ray film. The film was allowed to air dry and the calculations were made using ImageJ software. Briefly, each band was selected separately, and the software plotted the samples as peaks and gave the exact area of each peak. The expression of each target protein was normalized to β-actin level of the same animal.

### 4.9. Statistical Analyses

Data from behavioral tests were analyzed using two-way repeated measures ANOVA followed by Sidak’s multiple comparisons test and Mann–Whitney U-test. One-way ANOVA followed by Tukey’s multiple comparisons test was used to analyze the effects of BCP on cytokines mRNA expression. In the case of nonparametric data (*Tnf* in the brain), Kruskal–Wallis test followed by Dunn’s multiple comparisons test was used instead. Student’s *t*-test, Mann–Whitney U-test and one-way ANOVA followed by Tukey’s multiple comparisons test were used to analyze protein expression. Data were analyzed using GraphPad Prism 8.3.0 for Windows (GraphPad Software, La Jolla, CA, USA) and were expressed as the mean ± SEM for normally distributed data or median and interquartile range for skewed data. The differences were considered significant at *p* < 0.05.

## Figures and Tables

**Table 1:** Primer sequences of cyclophilin and cytokines.

Gene	Polarity	
	Sense Sequence 5′–3′	Anti-Sense Sequence 5′–3′
*Ppia* (cyclophilin A)	GCTTTTCGCCGCTTGCT	CTCGTCATCGGCCGTGAT
*Ifng* (interferon gamma)	ACAATGAACGCTACACACTGCAT	TGGCAGTAACAGCCAGAAACA
*Il1b* (interleukin 1 beta	TGGTGTGTGACGTTCCCATT	CAGCACGAGGCTTTTTTGTTG
*Tnf* (tumor necrosis factor alpha)	GGCTGCCCCGACTACGT	GACTTTCTCCTGGTATGAGATAGCAAA
